# Fengreqing Oral Liquid Exerts Anti-Inflammatory Effects by Promoting Apoptosis and Inhibiting PI3K/AKT and NF-κB Signaling Pathways

**DOI:** 10.3389/fphar.2022.824579

**Published:** 2022-03-14

**Authors:** Zhili Rao, Xiangyu Li, Xia Zhang, Jiuseng Zeng, Baojun Wang, Ruocong Yang, Nan Zeng

**Affiliations:** State Key Laboratory of Southwestern Chinese Medicine Resources, School of Pharmacy, Chengdu University of TCM, Chengdu, China

**Keywords:** Fengreqing oral liquid, anti-inflammation, apoptosis, acute lung injury, RAW 246.7 cells

## Abstract

Fengreqing oral liquid (FOL), a Chinese patent drug frequently used in clinical practice in China, is effective in treating inflammatory diseases of the upper respiratory tract such as colds and flu. However, its anti-inflammatory effects and mechanisms remain to be elucidated. In this study, the anti-inflammatory effects of FOL and its mechanisms on PI3K/AKT and NF-κB signaling pathways in LPS-induced RAW264.7 cells were explored, as well as the regulatory effect of FOL on apoptosis. In addition, the potential of FOL for the treatment of acute lung injury was explored in LPS-induced ALI mice. The results showed that treatment with FOL significantly reduced the levels of interleukin 1β (IL-1β), interleukin 6 (IL-6), nitric oxide (NO), and tumor necrosis factor α (TNF-α) in the supernatant of LPS-induced RAW264.7 cells, and also significantly reduced the phosphorylated protein levels of PI3K and AKT in the PI3K/AKT signaling pathway and also protein levels of NF-κB p50, phosphorylated NF-κB p65, and IκBα in the NF-κB signaling pathway. In addition, the results showed that FOL induced apoptosis in LPS-induced RAW264.7 cells at the level of 80%–90%, and significantly increased the protein expression levels of the pro-apoptotic Bax and cleaved-caspase-3. In LPS-induced ALI mice, FOL administration showed inhibition of IL-1β, IL-6, and TNF-α in Bronchoalveolar lavage fluid (BALF) and decreased protein expression levels of PI3K, AKT, NF-κB p50, and NF-κB p65, and elevated protein expression levels of Bax and cleaved-caspase-3 significantly. These results suggest that FOL may exert anti-inflammatory effects by inhibiting the PI3K/AKT signaling pathway to promote apoptosis and leading to attenuated activation of the NF-κB signaling pathway.

## Introduction

Fengreqing oral liquid (FOL), a Chinese patent medicine frequently used in clinical practice in China. In the theory of traditional Chinese medicine, it has the function of clearing away heat and toxic materials, expectorating, promoting the dispersing function of the lung, and expelling pathogenic factors from the exterior. Thus, it is mainly used for the treatment of fever, chills, headache, cough, runny nose, thirst, sore throat, and acute upper respiratory tract infections, which has a great curative effect (Jie et al., 2021; Ruocong et al., 2021). FOL combines two ancient Chinese medical formulas, Qingdai Decoction in *Songfeng Shuoyi (On Pestilence Recorded by Song Feng)*, a TCM classic written by Liu Kui in the Qing Dynasty, and Jiegeng Decoction in *Shanghanlun (Treatise on Cold Damage)*, a TCM Canon written by Zhang Zhongjin and considered as the source of all prescription mannuals. These two formulas have been used in China for thousands of years and are highly effective for the treatment of inflammatory respiratory diseases. Recent studies have investigated the pharmacological effects of FOL and found that it had a significant inhibitory effect on xylene-induced ear swelling in mice (Kaixi et al., 2021b). A further study found that it significantly reduced tumor necrosis factor-α (TNF-α) and histamine content and obviously increased interleukin-4 (IL-4) content in swollen tissues of mice ears. In addition, the mechanism of analgesic effect of FOL may be related to the reduction of prostaglandin E2 and bradykinin levels and the increase of β-endorphin level in serum of mice (Kaixi et al., 2021b). Additional studies have shown that FOL has antiviral and antibacterial activity both *in vitro* and *in vivo*. It has also been reported that FOL enhances the immune function of immunocompromised mice by improving nonspecific immunity, humoral immunity, and cellular immunity (Kaixi et al., 2021a; Jie et al., 2021).

Acute lung injury (ALI) is a life-threatening disease with high morbidity and mortality rates that is characterized by severe inflammatory responses and tissue damage in the lungs (Mokra and Kosutova, 2015; Qu et al., 2019a). The main pathologies of ALI include diffuse alveolar injury, lung edema formation, neutrophil-derived inflammation, and surfactant dysfunction (Herold et al., 2013; Nieman et al., 2015). At present, it is well recognized that excessive-activated inflammatory response is the main cause of exacerbation of ALI or is causing multi-organ failure. Therefore, a novel strategy through suppressing the inflammatory cascade response in the lungs might be a promising therapy for the treatment of ALI (Rao et al., 2022).

It is well known that NF-κB regulates the expression of inflammation cytokine genes (Lambrou et al., 2020). The activation of NF-κB may lead to the release of proinflammatory cytokines (such as TNF-α, IL-6, and IL-1β), chemokines, and adhesion molecules, whereby enhancing the inflammatory response. Specifically, NF-κB signaling pathway activation is associated with the regulation of several signaling pathways, including apoptosis. Apoptosis, a programmed form of cell death, is a process actively selected through a series of pathways without causing an inflammatory response (Xu et al., 2019). Apoptosis plays an indispensable and irreplaceable role in both physiological and pathological conditions, while apoptotic dysfunction can lead to disease, and over-activated apoptosis may lead to disease exacerbation (Pietenpol and Stewart, 2002; García-Arriaza et al., 2017).

In our previous study, we predicted the potential targets of FOL *via* the network pharmacology approach and verified it by *in vitro* inflammation cells. The results showed that PI3K-AKT signaling transfer might be the key target in regulating the concentration of inflammation mediators of FOL in the Toll-like receptor signaling pathway. The vitro experiments confirmed that FOL may significantly reduce the levels of NO, IL-1β, IL-6, and TNF-α produced in LPS induced RAW264.7cells, which may be related to the regulation effect of FOL on PI3K-AKT pathway (Ruocong et al., 2021). In this study, we further investigated whether the anti-inflammatory effects of FOL were related to the regulation of NF-κB signaling pathway and apoptosis, and further evaluated its potential for the treatment of ALI.

## Materials and Methods

### Preparation of FOL

FOL was provided by Xinlu Pharmaceutical Co. Ltd (Lot 200303; Sichuan, China) and composed of Gan Cao (*Glycyrrhiza uralensis* Fisch. ex DC. (Fabaceae)), Jie Geng (*Platycodon grandiflorus* (Jacq.) A. DC. (Campanulaceae)), Qing Dai (*Baphicacanthus cusia* (Nees) Bremek. (Acanthaceae)), Shan Yin Hua (Lonicera hypoglauca Miq. (Caprifoliaceae)), Gua Lou Pi (*Trichosanthes kirilowii* Maxim. (Cucurbitaceae)) and Xiong Dan Fen (Pulvis fellis ursi, Bile obtained from artificially cultured *Selenaretos thibetanus* Cuvier. by painless drainage). Briefly, 50 g of Qing Dai was macerated with 80% ethanol for 24 h and the percolate was collected. 850 g of Shan Yin Hua was extracted by redistillation to collect its aromatic water. The 500 g of Jie Gen, 400 g of Gua Lou Pi and 200 g of Gan Cao were steeped in water for half an hour and then decoction twice and the obtained filtrate was combined and concentrated by reducing pressure to a relative density of 1.10–1.15 g/ml at 90°C. Then ethanol was added to the above extract to reach 80% alcohol content and filtered, and the filtrate was combined with the percolate of Qing Dai and concentrated under reduced pressure to evaporate the ethanol. Then add the aromatic water of Shan Yin Hua and 5 g of Xiong Dan Fen, and adjust the pH to 6.0~6.5 with 10% sodium hydroxide solution. Finally, the extract was filtered and was added water to 1000 ml to fix the volume, sterilize and stored at 4°C until administration. In vitro study, 2 ml of FOL was taken and centrifuged at 3500 r/min for 10 min. The supernatant obtained was filtered through a 0.2 μm nylon microporous membrane and then stored at −20°C.

### Determination of the Major Components of FOL by High-Performance Liquid Chromatography–Mass Spectrometry

The main chemical components of FOL were analyzed by high-performance liquid chromatography–mass spectrometry (HPLC-MS) (UltiMate 3000 LC, Orbitrap Elite; Thermo Scientific). In brief, FOL was centrifuged at 12,000 *g* and the supernatant was filtered at 0.22 μm and eluted with an Acclaim™ RSLC 120 C18 column (Thermo, Sichuan, China). The eluted components are fed into the mass spectrometer to collect the corresponding mass spectral information. The obtained mass spectral information was feature extracted and pre-processed using SIEVE software (Thermo, Sichuan, China), normalized, and edited into a two-dimensional data matrix including retention time, compound molecular weight, observed value (sample), and peak intensity using Excel 2010 software. The obtained compound mass spectral information was compared with the mzcloud and mzvault databases, with information matching >85% as the screening condition.

### Cell Culture

RAW264.7 cells were purchased from the Shanghai Cell Bank of the Chinese Academy of Sciences and cultured in complete Dulbecco’s modified Eagle’s medium (DMEM) containing 10% fetal bovine serum and 1% penicillin–streptomycin solution in an incubator at 37°C and 5% CO_2_.

### Animals

Male ICR mice (6 weeks old, 18–22 g) were obtained from Sichuan Dashuo Biotechnology Co. Ltd. (Sichuan, China) and given free access to water and a standard diet *ad libitum*. The mice were acclimated for 3 days before administration and housed in a controlled room with a constant temperature of 24 ± 2°C, relative humidity of 55 ± 10%, and a 12:12-h light:dark cycle. All animal studies were conducted in accordance with the guidelines of the Committee for Animal Care and Use of Laboratory Animals, College of Pharmacy, Chengdu University of Traditional Chinese Medicine (No. 20200320).

### Determination of the Effect of FOL on Cell Viability by CCK-8 Assay

In our previous study, the effect of FOL on the viability of RAW264.7 cells was explored (Ruocong et al., 2021). We repeated this process in the current study and obtained the same results. Briefly, RAW264.7 cells (1 × 10^5^ cells/ml) were uniformly seeded in a 96-well cell culture plate and incubated at 37°C and 5% CO_2_ for 12 h. The cells were cultured with different concentrations of FOL (0.25%–0.50%) for 6 h before 100 μl of CCK-8 (Cell Counting Kit-8 Kit, Lot C1101130; YEASEN, Shanghai, China) solution (100 μl of DMEM containing 10 μl of CCK-8) was added to each well and incubated for 3 h. Then, the optical density value of each well was detected at 450 nm.

### LPS Stimulation and Detection of NO, IL-6, IL-1β, and TNF-α in Culture Supernatant

RAW264.7 cells (1 × 10^6^ cells/ml) were equally seeded in 6-well cell culture plates and cultured for 24 h. The cultured cells were divided into 5 groups, including control group, 0.30% of FOL group, LPS (1 μg/ml) group, LPS+0.30% of FOL group, and LPS+0.15% of FOL group. After aspirating the culture solutions, each group was pretreated with the corresponding concentrations of FOL for 6 h, while the control group and the LPS group contained an equal volume of DMEM. After 6 h of treatment, the culture solutions were removed. Except for the control group and 0.30% of FOL group in which DMEM was added, the cells in other groups were cultured with 1.5 ml of DMEM containing LPS (1 μg/ml) for 12 h. The cell culture supernatant was collected, and then the content of nitric oxide (NO) was determined by a Total Nitric Oxide Assay Kit (Beyotime Biotechnology, Lot 090420201102; Shanghai, China). The levels of IL-6, IL-1β, and TNF-α in the cell culture supernatant were measured by using enzyme-linked immunosorbent assay kits (ExCell Biotechnology, Lot 22A140, 22A128, 22A128; Shanghai, China), according to the manufacturer’s instructions.

### Effect of FOL on Apoptosis in LPS-Induced Inflammation Model of RAW264.7 Cells

RAW264.7 cells were seeded in a 6-well plate at a concentration of 10^6^ cells/ml and divided into 5 groups: control group, 0.30% of FOL group, LPS group, 0.30% of FOL + LPS group, and 0.15% of FOL + LPS group. After each group was incubated with the corresponding concentration of drug for 6 h, LPS (1 μg/ml) was added to each group except the control group and 0.30% of FOL group for 12 h. Then, the cells were added to Annexin V-FITC and PI Staining Solution in an Annexin V-FITC/PI Apoptosis Detection Kit (Shanghai Yisheng Biotechnology Co. Lot 40302ES20, Shanghai, China), and the apoptotic cells were detected by flow cytometry according to the manufacturer’s instructions.

### To Investigate the Relationship Between the Anti-Inflammatory Effect of FOL and the Regulation of the PI3K/AKT Signaling Pathway by LY294002 and 740-YP

Our previous study predicted the potential targets of FOL via the approach of network pharmacology, and the results showed that PI3K-AKT signaling transfer might be the key part in regulating the concentration of inflammation cytokines. To investigate the relationship between the anti-inflammatory effect of FOL and the regulation of the PI3K/AKT signaling pathway, LY294002 (Selleck Biotech Technology Co. Lot S1105, USA), a PI3K inhibitor, and 740-YP (Shanghai TOPSCIENCE Biochemical Technology Co. Lot TQ0003, China), an activator of PI3K, were used in this study. Briefly, RAW264.7 cells (1 × 10^6^ cells/ml) were equally seeded in 6-well cell culture plates and cultured for 24 h. The cultured cells were added with 1.5 ml of LY294002 (20 μM) or 740-YP (10 μM) solution for 2 h, and the other groups contained an equal volume of DMEM. Then, each group was pretreated with the corresponding concentrations of FOL for 6 h. After 6 h of treatment, the culture solutions were removed and cultured with 1.5 ml of DMEM containing LPS (1 μg/ml) for 12 h. The cytokines in the supernatant and proteins of cell lysate were measured.

### LPS-Induced ALI and Administration

Mice were randomly divided into six groups: control group, sham-operation group, model group, LPS + FOL (12 ml/kg) group, LPS + FOL (6 ml/kg) group, and LPS + Dexamethasone (Sigma-Aldrich, Lot D4902, Shanghai, China) (LPS + DEX) (5 mg/kg) group. The mice in the LPS + FOL group (12 ml/kg, 6 ml/kg) were administered the corresponding concentrations of FOL by intragastric injection for 5 successive days, while the mice in the control group, sham-operation group, and model group were administered water instead. The mice in the LPS + DEX group were administered DEX dissolved in pure water. On day 6, after 12 h of fasting, all mice received their final dose of the drug and then anesthetized by intraperitoneal injection of sodium pentobarbital (1%, 50 mg/kg) and immobilized. A sterile injection needle (1.0 ml) was used to puncture the trachea, which was then instilled with LPS (100 μl, 5 mg/kg) to induce ALI, while the mice in the sham-operation group were given an equal volume of sterile saline instead. After 8 h of LPS infusion, the mice were sacrificed, and the BALF and the lungs were collected for detection.

### Detecting the Contents of IL-6, IL-1β, and TNF-α in BALF

After each mouse was sacrificed, a syringe with the tip cut off was inserted into the trachea and ligated. Then, 1 ml of saline was slowly injected into the syringe and lavaged three times to obtain the BALF. The BALF obtained was centrifuged at 3500 r/min for 10 min. The supernatant of BALF was separated out, and the contents of IL-6, IL-1β, and TNF-α in the BALF supernatant were measured by enzyme-linked immunosorbent assay kits (ExCell Biotechnology, Lot 22A05702, 22A02901 22A10608; Shanghai, China), according to the manufacturer’s instructions.

### The Content of Cells in BALF

After the supernatant of BALF was separated out, 1 ml of PBS was added to resuspension cells. Then, the number of cells in BALF were counted by a cell counting plate under an electron microscope.

### Western Blotting

Proteins from cells and animal tissues were obtained by lysis of RIPA lysate buffer (Beyotime, Chengdu, China) containing 1 mM of phenylmethylsulfonyl fluoride (PMSF) (Med Chem Express, China). The protein concentrations were quantified by using the BCA kit (Beyotime, Chengdu, China), and all samples were adjusted to the same concentration. Then, the protein samples were mixed with 5 × loading buffer and heated to 95°C for 5 min. Afterwards, the proteins (10–30 μg) were separated by SDS-PAGE and electrophoretically transferred onto polyvinylidene fluoride membranes, and then blocked with 5% skimmed milk. The membranes were labeled with β-Actin (13E5) Rabbit mAb (Cell Signaling Technology, Lot #4970, USA), PI3 Kinase p85 Antibody (Cell Signaling Technology, Lot # 4292, USA), Akt (pan) (11E7) Rabbit mAb (Cell Signaling Technology, Lot #4685, USA), Phospho-PI3 Kinase p85 (Tyr458)/p55 (Tyr199) Antibody (Cell Signaling Technology, Lot#4228, USA), Phospho-Akt (Thr308) Antibody (Cell Signaling Technology, Lot#9275, USA), Apoptosis-Bcl-2 Antibody [Abmart Pharmaceutical Technology (Shanghai) Co. Lot P10415, Shanghai, China], Apoptosis regulator Bax [Abmart Pharmaceutical Technology (Shanghai) Co. Lot Q07812, Shanghai, China], Cleaved-Caspase 3 (Asp175), p17 Antibody [Abmart Pharmaceutical Technology (Shanghai) Co. Lot MB0711, Shanghai, China], Caspase-3 Antibody (Cell Signaling Technology, Lot #9662, USA), NF-κB1 p105/p50 (D4P4D) Rabbit mAb (Cell Signaling Technology, Lot#13586, USA), IκBα Antibody (Cell Signaling Technology, Lot #9242, USA), Phospho-IκBα (Ser32) (14D4) Rabbit mAb (Cell Signaling Technology, Lot #2859, USA), NF-κB p65 (D14E12) XP^®^ Rabbit mAb (BSA and Azide Free) (Cell Signaling Technology, Lot #69994, USA), and Phospho-NF-κB p65 (Ser536) (93H1) Rabbit mAb (Cell Signaling Technology, Lot #3033, USA) antibody at 4°C overnight, and then incubated with anti-rabbit HRP-linked secondary antibody at room temperature for an hour. Antibody detections were performed using a ChemiDoc XRS+ (BioRad, Hercules, CA, United States) image analysis system.

### Statistical Methods

The data are presented as mean ± S.D. SPSS 19.0 software (SPSS, Chicago, IL, United States) was used to determine the one-way analysis of variance with Fisher’s protected least significant difference post hoc test to notarize the importance of multiple comparisons. The *p* < 0.05 was considered a statistically obvious difference, and the *p* < 0.01 was a significant difference.

## Results

### Effects of FOL on RAW264.7 Cell Viability by CCK-8 Assay

**FIGURE 1:**
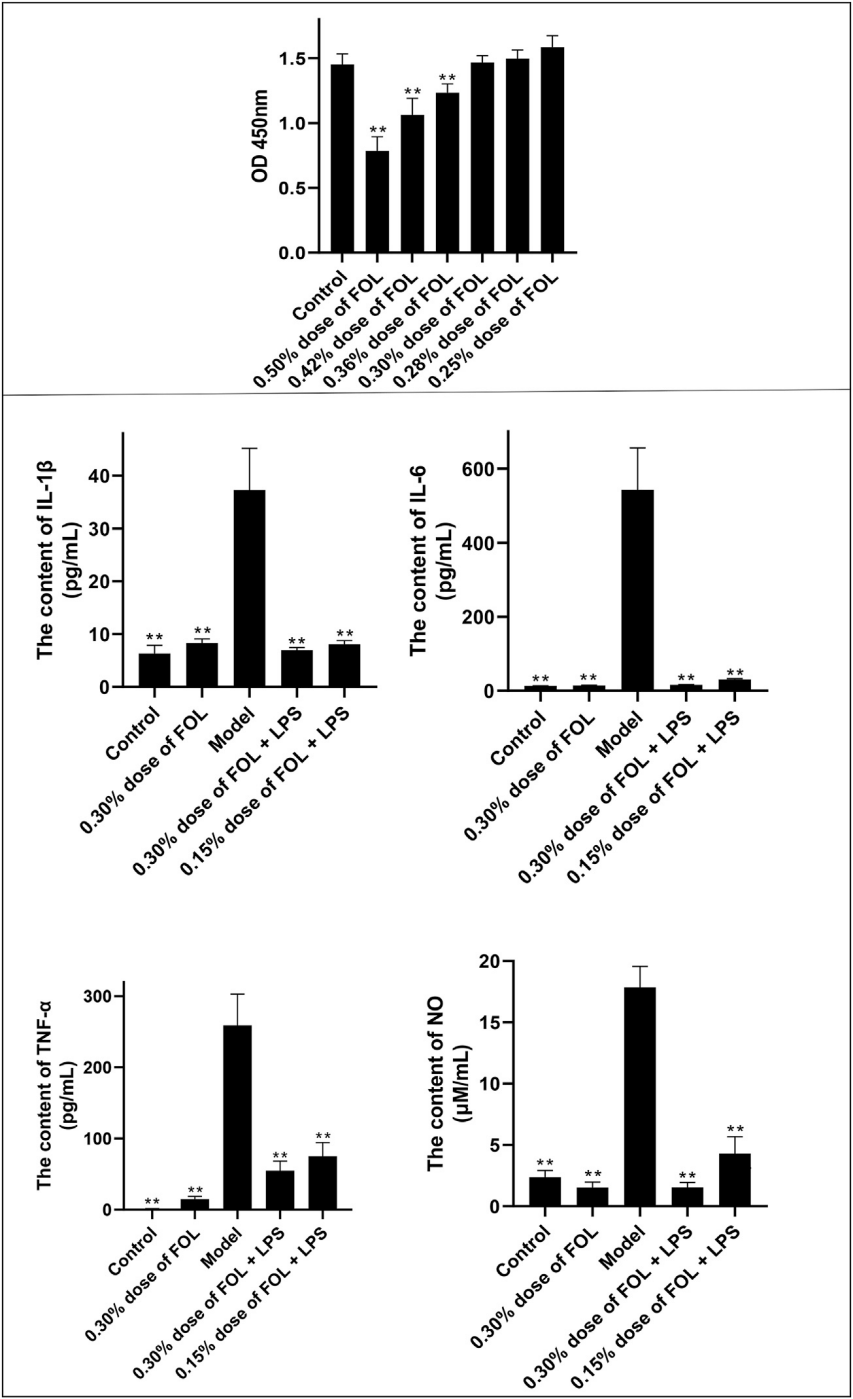
Anti-inflammatory effect of FOL in LPS-induced RAW264.7 cells. The effect of FOL on the viability of RAW264.7 cells was determined by CCK-8 assay; results showed that 0.30% or below of FOL did not affect the cell viability of RAW264.7, and the effects of FOL on the expression levels of IL-1β, IL-6, TNF-α, and NO in LPS-induced cell supernatants were determined by ELISA. Data are presented as the mean ± SD compared with the LPS-only control group. *p < 0.05; **p < 0.01 (*n* = 6).

The results showed that cells cultivated with 0.36%, 0.42%, and 0.50% of FOL for 6 h had a significant effect on RAW264.7 cell viability; the concentration of 0.30% and below had non-toxicity in RAW264.7 cells; therefore, the LPS-induced RAW264.7 cells were treated with 0.30% of FOL and 0.15% of FOL in the following experiments (Figure 1).

### Effects of FOL on Secreted Expression of NO, IL-6, IL-1β, and TNF-α in RAW264.7 Inflammatory Cells

As shown in Figure 1, compared with the control groups, the levels of NO, IL-6, IL-1β, and TNF-α in the cell culture supernatant of LPS group were upregulated significantly. In addition, the contents of NO, IL-1β, IL-6, and TNF-α in the supernatant of the cells pretreated with FOL (0.30% and 0.15%) for 6 h have been downregulated significantly (*p* < 0.01). These results indicate that FOL has anti-inflammatory effects on LPS-stimulated RAW264.7 cells.

### Effects of FOL on Inflammation *via* Inhibition of the PI3K/AKT Pathway

**FIGURE 2:**
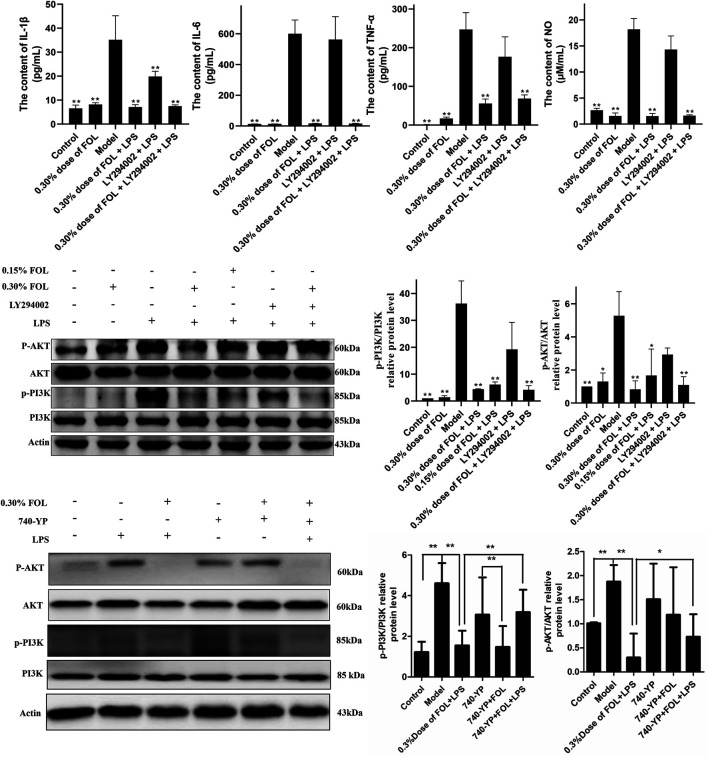
Effect of FOL on the PI3K/AKT signaling pathway in LPS-induced RAW264.7 cells. Inhibit/activate the PI3K/AKT signaling pathway with LY294002/740-YP prior to FOL administration to determine whether FOL inhibits inflammatory cytokine expression by modulating the PI3K/AKT signaling pathway. The effect of FOL on phosphorylation of PI3K and AKT protein was also detected by Western blotting. Data are presented as the mean ± SD compared with the LPS-only control group. *p < 0.05; **p < 0.01 (*n* = 6).

The results described above indicate that FOL can reduce the release of inflammatory cytokines. The PI3K/AKT signaling pathway may play a significant role in the release of pro-inflammatory cytokines. The results showed that 20 μM of LY294002 could only reduce the expression of IL-1β and have no effect on the secretion of IL-6, NO, and TNF-α in the LPS group. In addition, pretreatment with 20 μM LY294002 for 2 hours followed by treatment with 0.30% dose of FOL did not show significant differences in the secretion of IL-1β, IL-6, TNF-α, and NO compared to treatment with 0.30% dose of FOL alone. What is more, the protein levels of p-PI3K/PI3K and *p*-AKT/AKT were detected and the results showed that the protein levels of p-PI3K/PI3K and *p*-AKT/AKT were obviously increased in the LPS group (*p* < 0.01). It was found that 0.30% and 0.15% dose of FOL could decrease the protein levels of p-PI3K/PI3K and *p*-AKT/AKT in LPS-induced RAW264.7 inflammatory cells significantly (*p* < 0.01) (Figure 2). In addition, the PI3K agonist 740-YP significantly increased the expression levels of phosphorylated PI3K (p-PI3K) protein, while FOL inhibited PI3K activation induced by 740-YP. Meanwhile, in FOL-treated LPS-induced inflammatory cells, the inhibiting effect of FOL on phosphorylated PI3K and AKT was similarly attenuated by administration of 740-YP (*p* < 0.05).

### FOL Suppresses Inflammatory Factor Expression by Inhibiting NF-κB Signaling Pathway Activation

**FIGURE 3:**
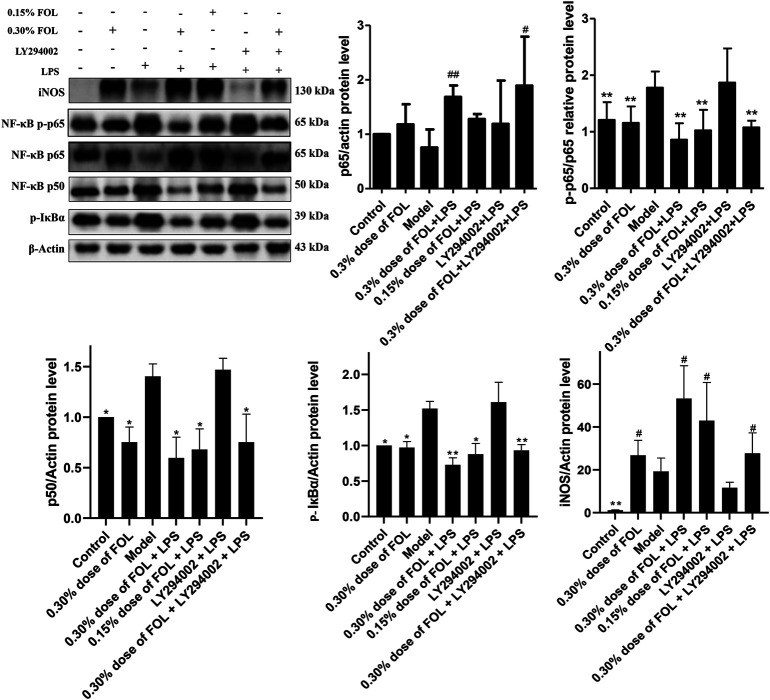
Effect of FOL on the NF-κB signaling pathway in LPS-induced RAW264.7 cells. The effects of FOL on protein expression levels of iNOS, NF-κB p65, NF-κB p50, and phosphorylated IκBα and NF-κB p-p65 were detected by Western blotting in LPS-induced RAW264.7 cells. Data are presented as the mean ± SD compared with the LPS-only control group. *p < 0.05; **p < 0.01 (*n* = 6).

The activation of the NF-κB signaling pathway is associated with the massive production of inflammatory cytokines, and the expression of key proteins of the NF-κB signaling pathway such as phosphorylated NF-κB p65, NF-κB p65, NF-κB p50, *p*-IκBα, and iNOS in LPS-induced RAW264.7cells was detected to investigate the regulatory effect of FOL on the NF-κB signaling pathway. The results showed that the NF-κB p50 and phosphorylated levels of NF-κB p65 and IκBα were significantly increased in LPS-induced RAW264.7 cells compared with the control group. In addition, the phosphorylated levels of NF-κB p65 were significantly decreased after 0.30% and 0.15% doses of FOL treatment, as well as the expression levels of NF-κB p50 and IκBα protein. Notably, the expression of iNOS was significantly increased in LPS-treated cells compared with controls, but the protein expression level of iNOS was further significantly increased after treatment with FOL. Meanwhile, LY294002 did not seem to show effective inhibition of NF-κB signaling pathway proteins such as NF-κB p65, NF-κB p65, NF-κB p50, and *p*-IκBα (Figure 3).

### Effect of FOL on Apoptosis in LPS-Induced Raw264.7 Cells

**FIGURE 4:**
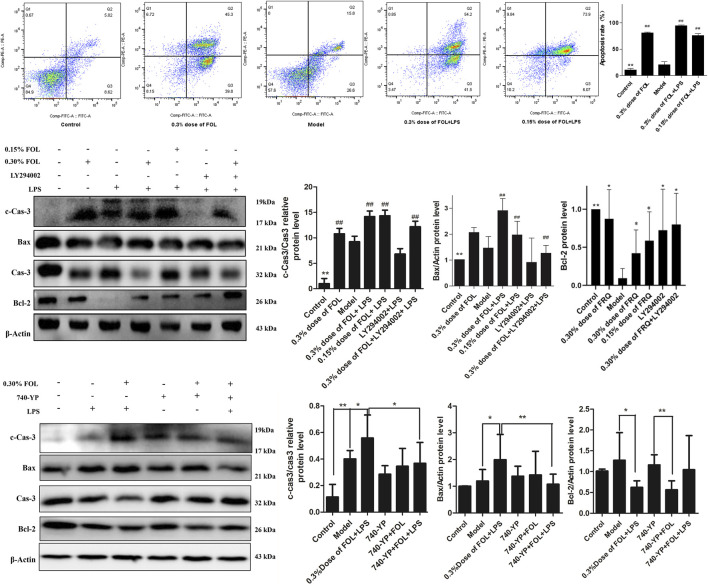
Effect of FOL on apoptosis in LPS induced RAW264.7 cells. The effect of FOL on the apoptosis level of RAW264.7 cells was detected by flow cytometry. The effects of FOL on the expression levels of apoptosis-related proteins caspase-3, cleaved-caspase-3, and Bax and BCL-2 proteins were detected by Western blotting. Data are presented as the mean ± SD compared with the LPS-only control group. *p < 0.05; **p < 0.01; *#p* < 0.05; *##p* < 0.01 (*n* = 6).

To examine the regulatory effect of FOL on apoptosis, the apoptosis level of RAW264.7 cells was examined by flow cytometry. The results found that treatment with FOL could significantly increase the apoptosis level of RAW264.7 cells. Treatment with 0.30% dose of FOL for 3 h resulted in apoptosis levels of normal cells being 80.83%. The level of apoptosis in the 0.30% of FOL + LPS group and the 0.15% of FOL + LPS group was 94.77% and 75.74%, respectively. In addition, FOL significantly increased the expression of the pro-apoptotic protein Bax, and also significantly increased the activation level of the apoptosis executive protein caspase-3 (Figure 4). In addition, pretreatment with the PI3K agonist 740-YP significantly reduced FOL-induced activation of caspase-3 and Bax.

### Main Chemical Components in FOL

**FIGURE 5:**
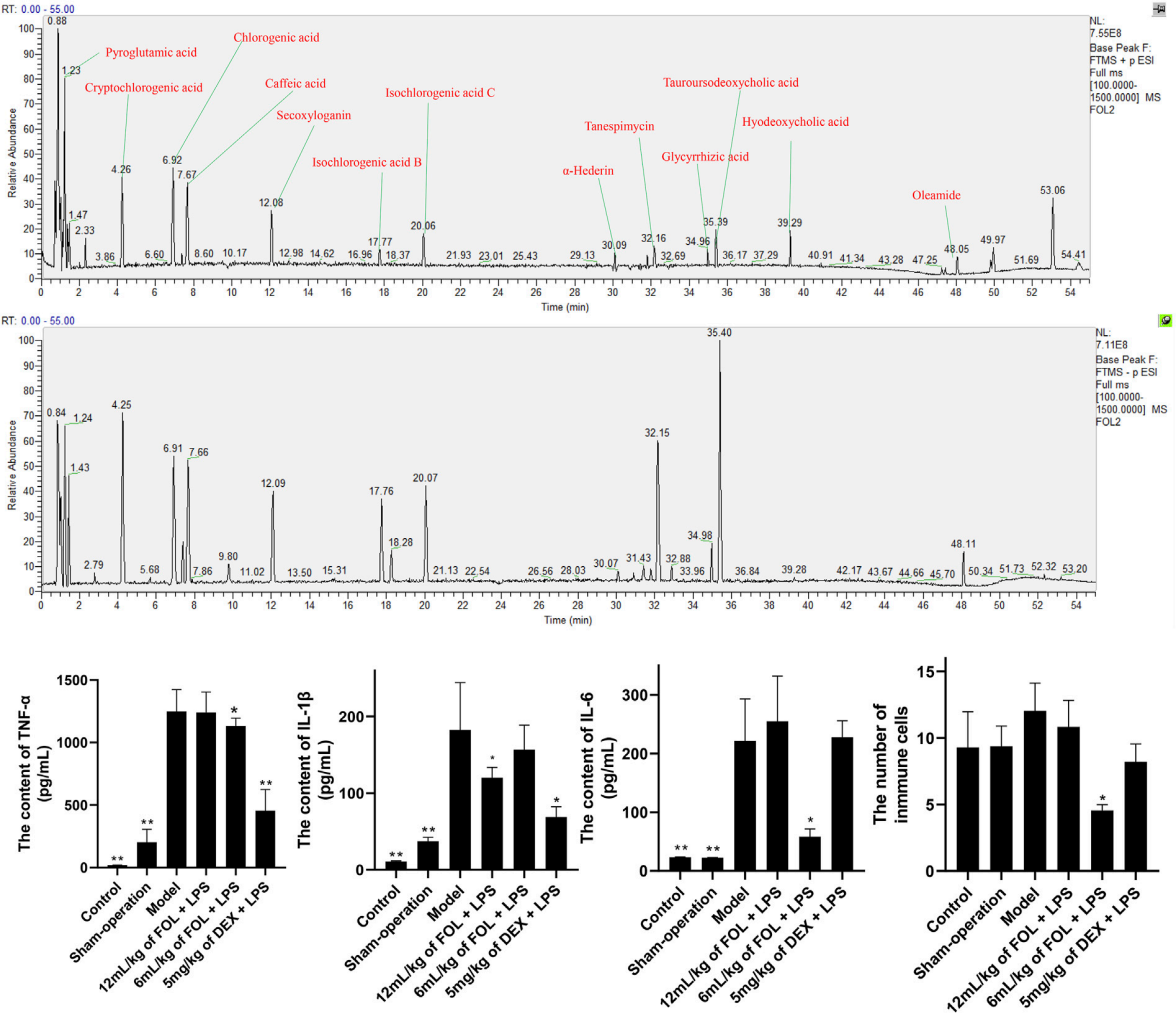
The main compounds in FOL and the effects of FOL on the expression levels of IL-6, IL-1β, and TNF-α in BALF of ALI mice. ALI mice were induced by tracheal drip of LPS; BALF was collected; the contents of IL-6, IL-1β, and TNF-α in BALF were measured by ELISA; and the number of cells in BALF was counted by microscopy. Data are presented as the mean ± SD compared with the model-only control group. *p < 0.05; **p < 0.01 (*n* = 6–9).

In view of the good anti-inflammatory effect demonstrated by FOL in *in vitro* experiments, we further analyzed the main components in FOL by HPLC-MS. The ESI ± chromatograms are shown in Figure 5. By comparing and analyzing with the mzcloud and mzvalut database, we found that the chemical components with a high content in FOL include pyroglutamic acid, cryptochlorogenic acid, chlorogenic acid, caffeic acid, secoxyloganin, isochlorogenic acid B, isochlorogenic acid C, α-hederin, tanespimycin, glycyrrhizic acid, tauroursodeoxycholic acid, hyodeoxycholic acid, and oleamide. The relative percentages of the top 30 compounds are shown in Table 1.

**TABLE 1:** The main chemical components in FOL.

NO.	Name	Formula	Molecular weight	RT [min]	Area (Max.)
1	Chlorogenic acid	C_16_H_18_O_9_	354.09512	6.918	8965166431
2	Tauroursodeoxycholic acid	C_26_H_45_NO_6_S	499.29715	35.396	7166230491
3	Cryptochlorogenic acid	C_16_H_18_O_9_	354.0951	4.25	6634482676
4	Secoxyloganin	C_17_H_24_O_11_	404.13205	12.083	6188063882
5	Cryptochlorogenic acid	C_16_H_18_O_9_	354.09509	7.666	5768446177
6	Isochlorogenic acid C	C_25_H_24_O_12_	516.12694	20.063	5591545088
7	Isochlorogenic acid B	C_25_H_24_O_12_	516.12693	17.757	4381799389
8	L-Pyroglutamic acid	C_5_H_7_NO_3_	129.04297	1.229	2517905360
9	Caffeic acid	C_9_H_8_O_4_	180.04243	7.667	2473003990
10	3,5-Dicaffeoylquinic acid	C_25_H_24_O_12_	516.12698	18.267	2443562323
11	Pyroglutamic acid	C_5_H_7_NO_3_	129.04181	1.231	1905452321
12	Quinic acid	C_7_H_12_O_6_	192.06295	0.902	1663621927
13	Gluconic acid	C_6_H_12_O_7_	136.03666	0.863	1513125519
14	Succinic acid	C_4_H_6_O_4_	118.02579	1.438	1365688355
15	2-(2-Acetyl-3,5-dihydroxyphenyl)acetic acid	C_10_H_10_O_5_	210.05304	12.082	1350152380
16	Wilforlide A	C_30_H_46_O_3_	454.3449	30.091	1345418190
17	Glycyrrhizic acid	C_42_H_62_O_16_	840.41457	34.975	1118412349
18	Trigonelline	C_7_H_7_NO_2_	137.04787	0.881	878398957.4
19	18-β-Glycyrrhetinic acid	C_30_H_46_O_4_	470.33973	34.967	655461577.8
20	Caffeic acid	C_9_H_8_O_4_	180.04247	20.064	622852246.7
21	Isoliquiritigenin	C_15_H_12_O_4_	256.07402	14.849	607038615.6
22	Oleamide	C_18_H_35_NO	281.27213	48.058	505622534.2
23	Isoliquiritigenin	C_15_H_12_O_4_	256.07402	15.19	493002608.5
24	Liguiritigenin-7-O-β-D-apiosyl-4′-O-β-D-glucoside	C_26_H_30_O_13_	550.16923	15.206	408566018.8
25	Liquiritin	C_21_H_22_O_9_	418.12676	14.85	402480550.4
26	D-Raffinose	C_18_H_32_O_16_	526.15178	0.886	353507621.3
27	Formononetin	C_16_H_12_O_4_	268.07383	33.188	326733854.5
28	Hyodeoxycholic acid	C_24_H_40_O_4_	392.29285	39.297	320472298.1
29	Diammonium glycyrrhizinate	C_42_H_62_O_16_	822.40396	35.763	290399140.7
30	Ethyl caffeate	C_11_H_12_O_4_	208.07337	24.747	275746054.3

### Effect of FOL on Inflammatory Cytokines in the BALF of LPS-Induced Acute Lung Injury Mice

As shown in Figure 5, the expression levels of IL-6, IL-1β, and TNF-α, and the total number of cells in BALF of LPS-induced ALI mice were measured. The results showed that the expression levels of IL-6, IL-1β, and TNF-α in the BALF of ALI mice were obviously higher than those of control and sham-operation mice. In addition, administration of FOL (6 ml/kg) could decrease the levels of IL-6 and TNF-α and the total number of cells in BALF of ALI mice significantly, and 12 ml/kg of FOL could significantly downregulate the level of IL-1β (*p* < 0.05). These results show that FOL can inhibit the release of IL-6, IL-1β, and TNF-α in ALI mice.

### Effect of FOL on PI3K/AKT and NF-κB Signaling Pathways in LPS-Induced ALI Mice

**FIGURE 6:**
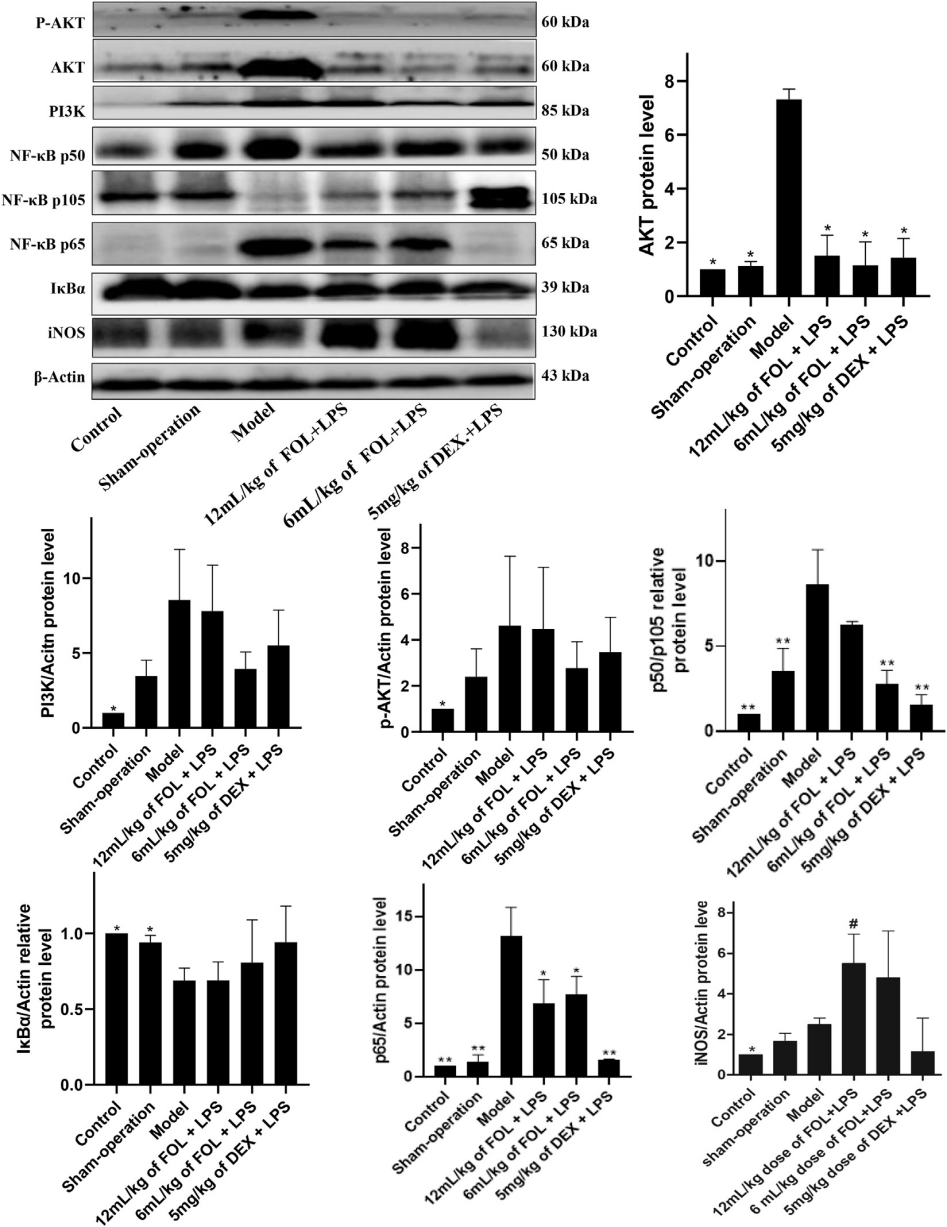
Effect of FOL on PI3K/AKT and NF-κB signaling pathways in lung tissues of LPS-induced ALI mice. The main chemical components in FOL were determined by high-performance liquid chromatography–mass spectrometry (HPLC-MS). The protein expression levels of AKT, PI3K, NF-κB p50, NF-κB p105, NF-κB p65, IκBα, iNOS, and phosphorylated protein of AKT in lung tissues of LPS-induced ALI mice were detected by Western blotting. Data are presented as the mean ± SD compared with the model-only control group. *p < 0.05; **p < 0.01 (*n* = 6–9).

As shown in Figure 6, the effects of FOL on the expression levels of proteins PI3K, AKT, and phosphorylated AKT in the lung tissue of LPS-induced ALI mice were examined. It was found that the protein expression levels of AKT, PI3K, and phosphorylated AKT were upregulated obviously in ALI mice (*p* < 0.05), and administration of FOL could reduce the protein expression levels of AKT significantly. In addition, the effect of FOL on the expression levels of key proteins of the NF-κB signaling pathway such as NF-κB p65, IκBα, iNOS, and the ratio of NF-κB p50 to NF-κB p105 in the lung tissue of LPS-induced ALI mice was also examined. The results showed that the expression levels of NF-κB p65, iNOS, and the ratio of NF-κB p50 to NF-κB p105 in LPS-induced ALI mice were increased significantly, and the expression level of IκBα was decreased; administration of FOL showed an inhibiting effect on the ratio of NF-κB p50 to NF-κB p105 as well as the expression level of NF-κB p65. In addition, FOL also significantly elevated iNOS expression in the lung tissues of LPS-induced ALI mice.

### Regulated Effects of FOL on Apoptosis in LPS-Induced ALI Mice

**FIGURE 7:**
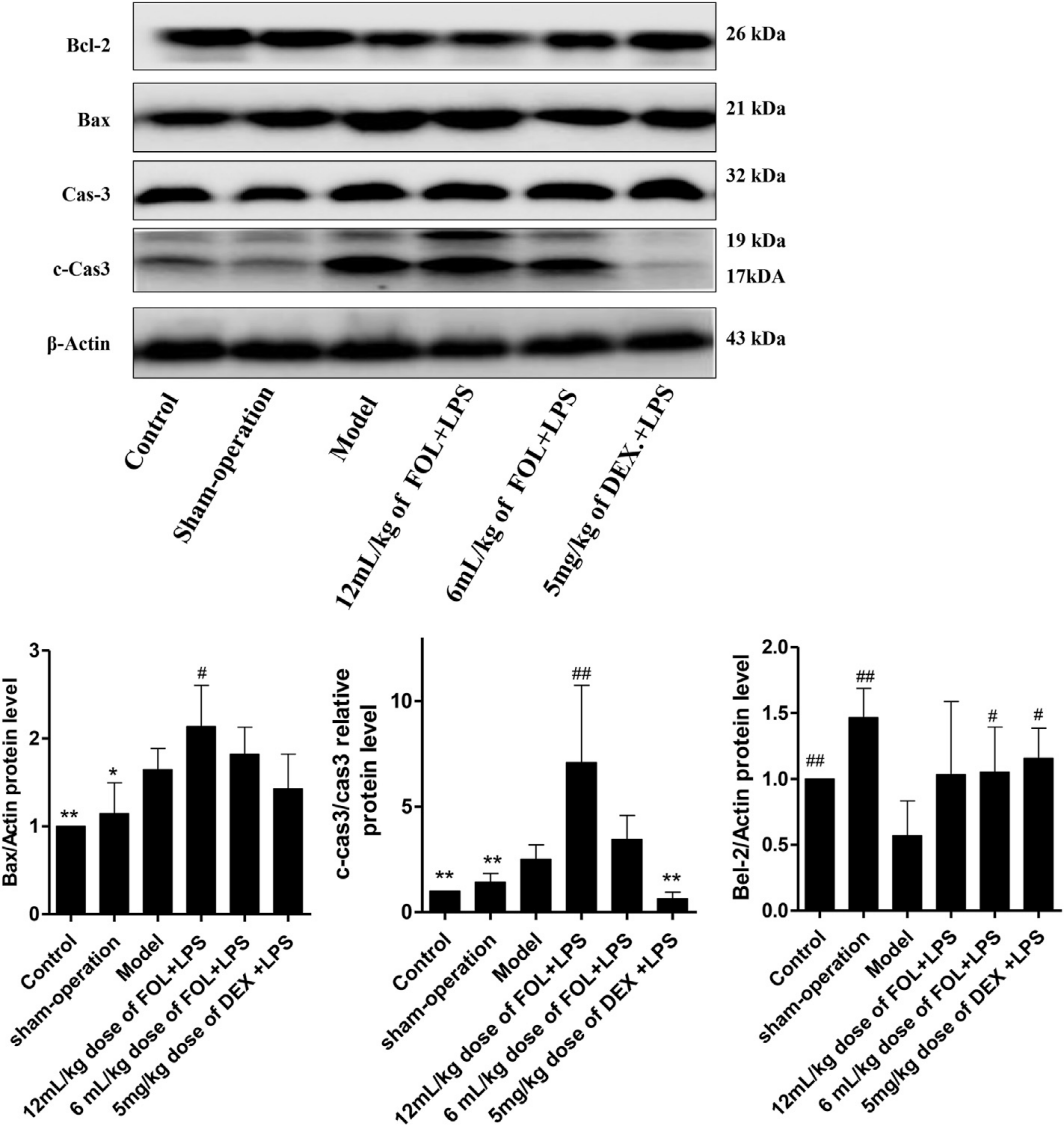
Effect of FOL on apoptosis in lungs of LPS-induced ALI mice. The effects of FOL on the expression levels of apoptosis-related proteins of caspase-3, cleaved-caspase-3, and Bax and Bcl-2 were detected by Western blotting. Data are presented as the mean ± SD compared with the LPS-only control group. *p < 0.05; **p < 0.01; *#p* < 0.05; *##p* < 0.01 (*n* = 6–9).

The protein expression levels of Bcl2, Bax, Caspase3, and cleaved-caspase 3 (c-caspase-3) were detected to investigate the regulated effects of FOL on apoptosis in LPS-induced ALI mice. As shown in Figure 7, compared with the control and sham-operated groups, the results showed that the expression of the pro-apoptotic protein Bax was significantly increased and the expression of the anti-apoptotic protein Bcl-2 was significantly decreased in LPS-induced ALI mice (*p* < 0.05). Furthermore, the ratio of c-Caspase3 to Caspase3 was also increased significantly. In comparison with LPS-induced ALI mice, Bax and c-Caspase3 were further increased in lung tissues of mice administration with a 12 ml/kg dose of FOL. This suggests that LPS-induced apoptosis was activated in mouse lung tissue, and treatment with FOL further exacerbated the level of apoptosis in lung tissue cells.

## Discussion

In the present study, we examined the anti-inflammatory effects of FOL in LPS-induced RAW264.7 cells and revealed the regulatory effects of FOL on apoptosis, PI3K/AKT, and NF-κB signaling pathways. In addition, the potential of FOL for the treatment of ALI was explored. FOL is widely used in China, and its clinical practice has been found to be effective in treating upper respiratory tract infections, effectively suppressing the level of inflammation in infected patients (Congxiao, 2020). Some studies have been conducted to evaluate the anti-inflammatory effect and mechanism of FOL. The results showed that FOL had an inhibitory effect on ear swelling in xylene ear swelling model mice, and reduced the levels of TNF-α and histamine and increased the levels of IL-4 in mouse ear tissues (Kaixi et al., 2021b). In addition, FOL reduced the number of torsions and serum levels of prostaglandin E2 and β-endorphin in acetic acid-induced torsion mice. FOL-treated mice showed prolonged foot-licking latency, a significant increase in serum β-EP content, and a significant decrease in PGE2 and bradykinin content (Kaixi et al., 2021b). These results show that FOL has a significant anti-inflammatory effect. In the present study, we found that FOL significantly reduced the levels of IL-6, IL-1β, TNF-α, and NO in the supernatant of LPS-induced RAW264.7 cells. It also reduced the concentration of IL-1β, IL-6, and TNF-α in BALF of LPS-induced ALI mice. These results further demonstrate the anti-inflammatory potential of FOL. However, the mechanism of the anti-inflammatory effect of FOL is still not elucidated.

In our previous study, the strategies and methods of network pharmacology were used to explore the characteristics and anti-inflammation effect of FOL and further verify it through an *in vitro* model (Ruocong et al., 2021). Through the enrichment and mapping analysis of network pharmacology, it is found that the PI3K-AKT pathway is the most critical intracellular signal transduction part of FOL intervention in wind-heat pattern. The PI3K/AKT signaling pathway is one of the important pathways associated with the regulation of cell survival in mammals that control cell growth, migration, proliferation, and metabolism (Pompura and Dominguez-Villar, 2018). The PI3K/AKT signaling pathway regulates a variety of cellular biological processes, including apoptosis, autophagy, inflammation, and oxidative stress (Du et al., 2017; Jiang et al., 2019; Shi et al., 2019). In this study, the LPS-induced ALI mice and LPS-induced RAW264.7 cells were implemented to validate the regulatory effects of FOL on the PI3K/AKT signaling pathway. The results showed that FOL exhibited inhibition of PI3K/AKT signaling pathway activation. In addition, FOL significantly inhibited PI3K phosphorylation activated by the PI3K agonist 740-YP. This information further confirmed the inhibitory effect of FOL on the PI3K/AKT signaling pathway. Notably, we used LY294002, a PI3K inhibitor shown to significantly inhibit PI3K and AKT activation, to further confirm the relationship between the anti-inflammatory effect of FOL and inhibition of the PI3K/AKT signaling pathway. However, the results showed that LY294002 could only reduce the content of IL-1β in the supernatant of LPS-induced RAW264.7 cells. In addition, treatment with LY294002 prior to FOL therapy versus FOL alone did not demonstrate a difference in the inhibition of inflammatory cytokine release. These results suggest that there are additional and more important regulatory pathways in the mechanisms of inhibiting the release of inflammatory cytokines in addition to the PI3K/AKT signaling pathway.

The NF-κB pathway is an important pathway for the regulation and release of inflammatory cytokines (Vallabhapurapu and Karin, 2009). The classical NF-κB pathway responds to stimuli from different immune receptors and leads to rapid NF-κB activation and inflammatory cytokine release (Sun, 2017). This process requires IκBα phosphorylation and loss of inhibition of NF-κB p50, which then mediates the nuclear translocation of NF-κB p50 and NF-κB p65 (Israël, 2010). Therefore, we also examined the effect of FOL on proteins related to the NF-κB signaling pathway in LPS-induced RAW264.7 cells. The results showed that FOL significantly inhibited the phosphorylation level of IκBα, as well as the phosphorylation level of NF-κB p65 and the processing of NF-κB p50 in NF-κB p105 protein. In LPS-induced ALI, FOL administration also significantly reduced the protein expression levels of NF-κB p65 and NF-κB p50. These results suggest that the effect of FOL on reducing inflammatory cytokine release may be related to the inhibition of NF-κB activation.

In addition, PI3K/AKT inhibition may lead to increased levels of apoptosis (Yang et al., 2018; Sreenivasulu et al., 2020; Chen et al., 2021). We first examined the effect of FOL on LPS-induced apoptosis in RAW264.7 cells by flow cytometry and showed that even in the absence of LPS, FOL treatment resulted in a level of cell apoptosis at 80%, and in the presence of LPS induction, FOL treatment resulted in a level of apoptosis of 90.4%, while in the LPS group, the rate of apoptosis cells is about 41.2%. Further assays of apoptosis-related proteins such as Bax, caspase-3, and Bcl-2 revealed that treatment with FOL significantly increased the activation level of the apoptosis-executing protein caspase-3 and the expression level of the pro-apoptotic protein Bax. These results suggest that FOL has a certain induced apoptotic effect on macrophages. Apoptosis is known to be an active process and has anti-inflammatory properties (Henson and Bratton, 2013). These results suggest that the anti-inflammation effect of FOL may be associated with the promotion of macrophage apoptosis. This result was further validated in *in vivo* experiments, where FOL significantly increased caspase-3 activation in LPS-induced ALI, along with increased expression of the pro-apoptotic protein Bax. The promotion of apoptosis does play a role in inhibiting the release of inflammatory cytokines, but at the same time, excessive apoptosis may also aggravate ALI (Giordano et al., 2008; Chambers et al., 2018). Related studies have shown that apoptotic activation is already present in ALI, and over-activated apoptosis leads to alveolar capillary barrier dysfunction, which, in turn, leads to the development of pulmonary edema and pulmonary fibrosis (Gill et al., 2014; Gill et al., 2015). Therefore, we hypothesize that the anti-ALI effect of FOL at the 12 ml/kg dose is worse than that of 6 ml/kg *in vivo* because of the stronger pro-apoptotic effect of higher doses of FOL. Even though it has a significant inhibitory effect on the inflammatory response, current evidence suggests that it may exacerbate ALI because of its pro-apoptotic effects.

Compositional analysis revealed high levels of pyroglutamic acid, cryptochlorogenic acid, chlorogenic acid, caffeic acid, secoxyloganin, isochlorogenic acid B, isochlorogenic acid C, α-hederin, tanespimycin, glycyrrhizic acid, tauroursodeoxycholic acid, hyodeoxycholic acid, and oleamide in FOL. Cryptochlorogenic acid, caffeic acid, chlorogenic acid, isochlorogenic acid B, and isochlorogenic acid C are the main active substances in Shan Yin Hua. The literature shows that cryptochlorogenic acid dose-dependently inhibits the production of NO, TNF-α, and IL-6 in LPS-induced RAW264.7 cells and inhibits the nuclear translocation of NF-κB by blocking the phosphorylation of IκB kinase (IKK) and degrading IκB (Zhao et al., 2020). Caffeic acid is a natural compound with a variety of biological activities that can be metabolized from chlorogenic acid, one of the metabolites of chlorogenic acid (Touaibia et al., 2011) and it has good anti-microbial activity against microbial pathogens (Khan et al., 2021). It has been shown that caffeic acid can induce apoptosis by inhibiting the activity of Bcl-2 (Chang et al., 2010). Isochlorogenic acid is also a natural compound with strong biological activity; isochlorogenic acid A could reduce the LPS-induced ALI by inhibiting the activation of the Nf-κB/NLRP3 signaling pathway, and it also has the effect of promoting apoptosis (Puangpraphant et al., 2011; Wang and Xiao, 2019; Liu et al., 2020). α-Hederin, a monodesmosidic triterpenoid saponin, exhibits promising antitumor potential against a variety of human cancer cell lines, and has a definite apoptosis-promoting effect (Sun et al., 2018; Wang et al., 2019; Wang et al., 2020). Tanespimycin, an inhibitor of HSP90, was found to inhibit AKT phosphorylation and demonstrate synergistic pro-apoptotic effects in combination with Tipifarnib (Krzykowska-Petitjean et al., 2012). Glycyrrhetinic acid and glycyrrhizin are the active ingredients in *Glycyrrhiza uralensis* Fisch. (Gan Cao) and have many pharmacological activities, such as anti-inflammatory, anti-viral, and anti-tumor activities, as well as participation in immune regulation (Chen et al., 2020). Studies have shown that glycyrrhetinic acid demonstrates inhibition of PI3K/AKT activation in LPS-induced ALI, which is consistent with our results (Wang et al., 2011; Qu et al., 2019b). Tauroursodeoxycholic acid, an endogenous bile acid, is the main compound of Fel Selenarcti (Xiong Dan Fen) and a strong modulator of apoptosis in several cell types (Castro et al., 2004). Hyodeoxycholic acid is also the main compound of Fel Selenarcti (Xiong Dan Fen) and can suppress intestinal epithelial cell proliferation through inhibiting the FXR-PI3K/AKT pathway (Song et al., 2020). In conclusion, FOL contains a variety of natural active ingredients, some of which have been reported to promote apoptosis and have regulatory effects on PI3K/AKT and NF-κB signaling pathways. This is consistent with the results we have obtained above.

Another interesting result showed that FOL administration resulted in a significant increase in iNOS protein expression, which apparently contradicts the result that FOL reduces NO content in cell supernatants. In the literature, iNOS expression was found to be induced by appropriate stimuli (e.g., cytokines and hypoxia), resulting in the production of NO (Wilmes et al., 2020). In addition, iNOS is a homodimer with a molecular weight of about 130–135 kDa. It adopts a dual structural domain structure where the homodimerization process required for enzyme activation occurs in both oxygen domains, leading to rapid conformational changes and depending on the activity of L-arginine (Kielbik et al., 2019). Therefore, the result of reduced NO content by FOL is not related to the inhibition of iNOS protein expression, and the FOL may inhibit the biological pathway of NO synthesis by inhibiting the activity of iNOS. This deserves further investigation, because iNOS over-activation is often associated with inflammation and malignant diseases.

In conclusion, FOL has significant anti-inflammatory potential, and its anti-inflammatory mechanism may be related to its inhibition effect on the PI3K/AKT signaling pathway, which, in turn, activates apoptosis and thus inhibits NF-κB signaling pathway activation. Although FOL has good anti-inflammatory effects, its application in the treatment of ALI requires more in-depth evaluation and consideration.

## Data Availability

The original contributions presented in the study are included in the article/Supplementary Material, further inquiries can be directed to the corresponding author.

## References

[B1] Castro R. E. Solá S. Ramalho R. M. Steer C. J. Rodrigues C. M. (2004). The Bile Acid Tauroursodeoxycholic Acid Modulates Phosphorylation and Translocation of Bad via Phosphatidylinositol 3-Kinase in Glutamate-Induced Apoptosis of Rat Cortical Neurons. J. Pharmacol. Exp. Ther. 311, 845–852. 10.1124/jpet.104.070532 15190125

[B2] Chambers E. Rounds S. Lu Q. (2018). Pulmonary Endothelial Cell Apoptosis in Emphysema and Acute Lung Injury. Adv. Anat. Embryol. Cel. Biol. 228, 63–86. 10.1007/978-3-319-68483-3_4 PMC5930489 29288386

[B3] Chang W. C. Hsieh C. H. Hsiao M. W. Lin W. C. Hung Y. C. Ye J. C. (2010). Caffeic Acid Induces Apoptosis in Human Cervical Cancer Cells through the Mitochondrial Pathway. Taiwan J. Obstet. Gynecol. 49, 419–424. 10.1016/S1028-4559(10)60092-7 21199742

[B4] Chen K. Yang R. Shen F. Q. Zhu H. L. (2020). Advances in Pharmacological Activities and Mechanisms of Glycyrrhizic Acid. Curr. Med. Chem. 27, 6219–6243. 10.2174/0929867325666191011115407 31612817

[B5] Chen Y. H. Yang S. F. Yang C. K. Tsai H. D. Chen T. H. Chou M. C. (2021). Metformin Induces Apoptosis and Inhibits Migration by Activating the AMPK/p53 axis and Suppressing PI3K/AKT Signaling in Human Cervical Cancer Cells. Mol. Med. Rep. 23, 88. 10.3892/mmr.2020.11725 33236135 PMC7716426

[B6] Congxiao Z. (2020). Multicenter Clinical Trial of Xiaoer Fengreqing Mixture in Treatment of Acute Upper Respiratory Tract Infection (Anemopyretic Cold) in Children. Drug Eval. Res. 43, 2450–2456. 10.7501/j.issn.1674-6376.2020.12.017

[B7] Du C. Zhang T. Xiao X. Shi Y. Duan H. Ren Y. (2017). Protease-activated Receptor-2 Promotes Kidney Tubular Epithelial Inflammation by Inhibiting Autophagy via the PI3K/Akt/mTOR Signalling Pathway. Biochem. J. 474, 2733–2747. 10.1042/BCJ20170272 28694352

[B8] García-Arriaza J. Perdiguero B. Heeney J. L. Seaman M. S. Montefiori D. C. Yates N. L. (2017). HIV/AIDS Vaccine Candidates Based on Replication-Competent Recombinant Poxvirus NYVAC-C-KC Expressing Trimeric Gp140 and Gag-Derived Virus-like Particles or Lacking the Viral Molecule B19 that Inhibits Type I Interferon Activate Relevant HIV-1-Specific B and T Cell Immune Functions in Nonhuman Primates. J. Virol. 91, e02182. 10.1128/JVI.02182-16 28179536 PMC5391471

[B9] Gill S. E. Taneja R. Rohan M. Wang L. Mehta S. (2014). Pulmonary Microvascular Albumin Leak Is Associated with Endothelial Cell Death in Murine Sepsis-Induced Lung Injury *In Vivo* . PLoS One 9, e88501. 10.1371/journal.pone.0088501 24516666 PMC3917898

[B10] Gill S. E. Rohan M. Mehta S. (2015). Role of Pulmonary Microvascular Endothelial Cell Apoptosis in Murine Sepsis-Induced Lung Injury *In Vivo* . Respir. Res. 16, 109. 10.1186/s12931-015-0266-7 26376777 PMC4574190

[B11] Giordano R. J. Lahdenranta J. Zhen L. Chukwueke U. Petrache I. Langley R. R. (2008). Targeted Induction of Lung Endothelial Cell Apoptosis Causes Emphysema-like Changes in the Mouse. J. Biol. Chem. 283, 29447–29460. 10.1074/jbc.M804595200 18718906 PMC2570855

[B12] Henson P. M. Bratton D. L. (2013). Antiinflammatory Effects of Apoptotic Cells. J. Clin. Invest. 123, 2773–2774. 10.1172/JCI69344 23863635 PMC3696541

[B13] Herold S. Gabrielli N. M. Vadász I. (2013). Novel Concepts of Acute Lung Injury and Alveolar-Capillary Barrier Dysfunction. Am. J. Physiol. Lung Cel. Mol. Physiol. 305, L665–L681. 10.1152/ajplung.00232.2013 24039257

[B14] Israël A. (2010). The IKK Complex, a central Regulator of NF-kappaB Activation. Cold Spring Harb. Perspect. Biol. 2, a000158. 10.1101/cshperspect.a000158 20300203 PMC2829958

[B15] Jiang T. Zhang L. Ding M. Li M. (2019). Protective Effect of Vasicine against Myocardial Infarction in Rats via Modulation of Oxidative Stress, Inflammation, and the PI3K/Akt Pathway. Drug Des. Devel. Ther. 13, 3773–3784. 10.2147/DDDT.S220396 PMC6827513 31802850

[B16] Jie L. Yuzuo C. Kaixi O. Baoning W. Fu P. Chenghao Y. (2021). Anti-bacterial and Anti-viral Effects of Fengreqing Oral Liquid (风热清口服液) *In Vitro* and *In Vivo* . J. Tradit. Chin. Med. 41, 530–538. 10.19852/j.cnki.jtcm.2021.03.005 34392645

[B17] Kaixi O. Jie L. Yuan Z. Ziyi Z. Yuming Z. Chenghao Y. (2021a). Study on Immunomodulatory Property of Fengreqing Oral Liquid on Immunosuppressive Mice. World Chin. Med. 16, 2591–2595+2599. 10.3969/j.issn.1673-7202.2021.17.013

[B18] Kaixi O. Jie L. Yuming Z. Yuan Z. Ziyi Z. Chenghao Y. (2021b). Anti-inflammatory and Analgesic Effects of Fengreqing Oral Liquid and its Mechanism. Chin. J. Basic Med. Tradit. Chin. Med. 27, 1104–1107+1110. 10.19945/j.cnki.issn.1006-3250.2021.07.016

[B19] Khan F. Bamunuarachchi N. I. Tabassum N. Kim Y. M. (2021). Caffeic Acid and its Derivatives: Antimicrobial Drugs toward Microbial Pathogens. J. Agric. Food Chem. 69, 2979–3004. 10.1021/acs.jafc.0c07579 33656341

[B20] Kielbik M. Szulc-Kielbik I. Klink M. (2019). The Potential Role of iNOS in Ovarian Cancer Progression and Chemoresistance. Int. J. Mol. Sci. 20, 1751. 10.3390/ijms20071751 PMC6479373 30970628

[B21] Krzykowska-Petitjean K. Małecki J. Bentke A. Ostrowska B. Laidler P. (2012). Tipifarnib and Tanespimycin Show Synergic Proapoptotic Activity in U937 Cells. J. Cancer Res. Clin. Oncol. 138, 537–544. 10.1007/s00432-011-1131-9 22209975 PMC3278622

[B22] Lambrou G. I. Hatziagapiou K. Vlahopoulos S. (2020). Inflammation and Tissue Homeostasis: the NF-κB System in Physiology and Malignant Progression. Mol. Biol. Rep. 47, 4047–4063. 10.1007/s11033-020-05410-w 32239468

[B23] Liu Y. Wang Y. Liu M. Yang C. Cao F. Liang J. (2020). Isochlorogenic Acid A Inhibits Proliferation and Migration and Promotes Apoptosis in MH7A Cells Induced by TNF-α. Xi Bao Yu Fen Zi Mian Yi Xue Za Zhi 36, 693–698. 10.13423/j.cnki.cjcmi.009047 32958125

[B24] Mokra D. Kosutova P. (2015). Biomarkers in Acute Lung Injury. Respir. Physiol. Neurobiol. 209, 52–58. 10.1016/j.resp.2014.10.006 25466727

[B25] Nieman G. F. Gatto L. A. Habashi N. M. (2015). Impact of Mechanical Ventilation on the Pathophysiology of Progressive Acute Lung Injury. J. Appl. Physiol. (1985) 119, 1245–1261. 10.1152/japplphysiol.00659.2015 26472873

[B26] Pietenpol J. A. Stewart Z. A. (2002). Cell Cycle Checkpoint Signaling: Cell Cycle Arrest versus Apoptosis. Toxicology 181-182, 475–481. 10.1016/s0300-483x(02)00460-2 12505356

[B27] Pompura S. L. Dominguez-Villar M. (2018). The PI3K/AKT Signaling Pathway in Regulatory T-Cell Development, Stability, and Function. J. Leukoc. Biol. 103, 1065–1076. 10.1002/jlb.2mir0817-349r 29357116

[B28] Puangpraphant S. Berhow M. A. Vermillion K. Potts G. Gonzalez De Mejia E. (2011). Dicaffeoylquinic Acids in Yerba Mate (Ilex Paraguariensis St. Hilaire) Inhibit NF-κB Nucleus Translocation in Macrophages and Induce Apoptosis by Activating Caspases-8 and -3 in Human colon Cancer Cells. Mol. Nutr. Food Res. 55, 1509–1522. 10.1002/mnfr.201100128 21656672

[B29] Qu L. Chen C. Chen Y. Li Y. Tang F. Huang H. (2019a). High-Mobility Group Box 1 (HMGB1) and Autophagy in Acute Lung Injury (ALI): A Review. Med. Sci. Monit. 25, 1828–1837. 10.12659/MSM.912867 30853709 PMC6423734

[B30] Qu L. Chen C. He W. Chen Y. Li Y. Wen Y. (2019b). Glycyrrhizic Acid Ameliorates LPS-Induced Acute Lung Injury by Regulating Autophagy through the PI3K/AKT/mTOR Pathway. Am. J. Transl Res. 11, 2042–2055. 31105816 PMC6511780

[B47] Rao Z. Zeng J. Li X. Peng L. Wang B. Luan F. (2022). JFNE-A Isolated from Jing-Fang n-Butanol Extract Attenuates Lipopolysaccharide-Induced Acute Lung Injury by Inhibiting Oxidative Stress and the NF-κB Signaling Pathway via Promotion of Autophagy. Phytomed. 96, 153891. 10.1016/j.phymed.2021.153891 35026506

[B31] Ruocong Y. Zhili R. Xiangyu L. Dejian W. Xi P. Nan Z. (2021). Network Pharmacology and *In Vitro* Study of Fengreqing Oral Liquid (风热清口服液) in the Intervention of Wind-Heat Pattern. J. Tradit. Chin. Med. 41, 695–705. 10.19852/j.cnki.jtcm.2021.05.005 34708627

[B32] Shi J. Yu J. Zhang Y. Wu L. Dong S. Wu L. (2019). PI3K/Akt Pathway-Mediated HO-1 Induction Regulates Mitochondrial Quality Control and Attenuates Endotoxin-Induced Acute Lung Injury. Lab. Invest. 99, 1795–1809. 10.1038/s41374-019-0286-x 31570770

[B33] Song M. Yang Q. Zhang F. Chen L. Su H. Yang X. (2020). Hyodeoxycholic Acid (HDCA) Suppresses Intestinal Epithelial Cell Proliferation through FXR-PI3K/AKT Pathway, Accompanied by Alteration of Bile Acids Metabolism Profiles Induced by Gut Bacteria. Faseb J. 34, 7103–7117. 10.1096/fj.201903244R 32246800

[B34] Sreenivasulu K. Nandeesha H. Dorairajan L. N. Nachiappa Ganesh R. (2020). Over Expression of PI3K-AkT Reduces Apoptosis and Increases Prostate Size in Benign Prostatic Hyperplasia. Aging Male 23, 440–446. 10.1080/13685538.2018.1519014 30295140

[B35] Sun D. Shen W. Zhang F. Fan H. Tan J. Li L. (2018). α-Hederin Arrests Cell Cycle at G2/M Checkpoint and Promotes Mitochondrial Apoptosis by Blocking Nuclear Factor-κB Signaling in Colon Cancer Cells. Biomed. Res. Int. 2018, 2548378. 10.1155/2018/2548378 30363706 PMC6180961

[B36] Sun S. C. (2017). The Non-canonical NF-κB Pathway in Immunity and Inflammation. Nat. Rev. Immunol. 17, 545–558. 10.1038/nri.2017.52 28580957 PMC5753586

[B37] Touaibia M. Jean-François J. Doiron J. (2011). Caffeic Acid, a Versatile Pharmacophore: an Overview. Mini Rev. Med. Chem. 11, 695–713. 10.2174/138955711796268750 21679136

[B38] Vallabhapurapu S. Karin M. (2009). Regulation and Function of NF-kappaB Transcription Factors in the Immune System. Annu. Rev. Immunol. 27, 693–733. 10.1146/annurev.immunol.021908.132641 19302050

[B39] Wang Q. Xiao L. (2019). Isochlorogenic Acid A Attenuates Acute Lung Injury Induced by LPS via Nf-κB/NLRP3 Signaling Pathway. Am. J. Transl. Res. 11, 7018–7026. 31814905 PMC6895519

[B40] Wang C. Y. Kao T. C. Lo W. H. Yen G. C. (2011). Glycyrrhizic Acid and 18β-Glycyrrhetinic Acid Modulate Lipopolysaccharide-Induced Inflammatory Response by Suppression of NF-κB through PI3K P110δ and P110γ Inhibitions. J. Agric. Food Chem. 59, 7726–7733. 10.1021/jf2013265 21644799

[B41] Wang J. Wu D. Zhang J. Liu H. Wu J. Dong W. (2019). α-Hederin Induces Apoptosis of Esophageal Squamous Cell Carcinoma via an Oxidative and Mitochondrial-dependent Pathway. Dig. Dis. Sci. 64, 3528–3538. 10.1007/s10620-019-05689-1 31273592

[B42] Wang J. Deng H. Zhang J. Wu D. Li J. Ma J. (2020). α-Hederin Induces the Apoptosis of Gastric Cancer Cells Accompanied by Glutathione Decrement and Reactive Oxygen Species Generation via Activating Mitochondrial Dependent Pathway. Phytother Res. 34, 601–611. 10.1002/ptr.6548 31777126

[B43] Wilmes V. Scheiper S. Roehr W. Niess C. Kippenberger S. Steinhorst K. (2020). Increased Inducible Nitric Oxide Synthase (iNOS) Expression in Human Myocardial Infarction. Int. J. Leg. Med 134, 575–581. 10.1007/s00414-019-02051-y 30927077

[B44] Xu X. Lai Y. Hua Z. C. (2019). Apoptosis and Apoptotic Body: Disease Message and Therapeutic Target Potentials. Biosci. Rep. 39, BSR20180992. 10.1042/BSR20180992 30530866 PMC6340950

[B45] Yang J. Pi C. Wang G. (2018). Inhibition of PI3K/Akt/mTOR Pathway by Apigenin Induces Apoptosis and Autophagy in Hepatocellular Carcinoma Cells. Biomed. Pharmacother. 103, 699–707. 10.1016/j.biopha.2018.04.072 29680738

[B46] Zhao X. L. Yu L. Zhang S. D. Ping K. Ni H. Y. Qin X. Y. (2020). Cryptochlorogenic Acid Attenuates LPS-Induced Inflammatory Response and Oxidative Stress via Upregulation of the Nrf2/HO-1 Signaling Pathway in RAW 264.7 Macrophages. Int. Immunopharmacol. 83, 106436. 10.1016/j.intimp.2020.106436 32234671

